# Renal recovery after acute kidney injury: choice of initial renal replacement therapy modality still matters

**DOI:** 10.1186/cc13936

**Published:** 2014-06-20

**Authors:** Antoine G Schneider, Sean M Bagshaw

**Affiliations:** 1 Intensive Care Unit, Centre Hospitalo-Universitaire Vaudois, 46 rue du Bugnon, 1011 Lausanne, Switzerland; 2 Division of Critical Care Medicine, Faculty of Medicine and Dentistry, University of Alberta, 2-124E Clinical Sciences Building, 8440-112 Street NW, Edmonton, Alberta T6G 2B7, Canada

## Abstract

Renal replacement therapy can be applied either in an intermittent fashion or in a continuous fashion in severe acute kidney injury. To date, no modality has been shown to consistently improve patient survival. In the study recently reported by Sun and colleagues, continuous application of renal replacement therapy was associated with improved renal recovery, defined by lower risk of long-term need for chronic dialysis therapy. This association between nonrecovery and intermittent renal replacement therapy may be explained by a higher rate of hypotensive episodes and the lower capacity for fluid removal during the first 72 hours of therapy. Altogether, this study adds to the growing body of evidence to suggest improved likelihood of recovery of kidney function in critically ill survivors of AKI with continuous modalities for renal replacement therapy.

## 

In recent years, there has been increased interest in the long-term outcomes for patients who survive an episode of critical illness. For those survivors who experienced severe acute kidney injury (AKI) during the course of their critical illness, renal recovery is of upmost importance. Indeed, nonrecovery or incomplete recovery of renal function can translate into a need for long-term dialysis – a treatment associated with low quality of life and representing a major burden for healthcare systems.

In the previous issue of *Critical Care*, Sun and colleagues have compared the outcomes of 145 patients who required renal replacement therapy (RRT) for sepsis-related AKI [1]. Their findings suggest that recovery of kidney function to dialysis independence at 60 days was strongly associated with the initial RRT modality applied. Indeed, application of RRT in a continuous fashion (continuous venovenous hemodiafiltration (CVVHDF)) was associated with a higher rate of renal recovery than its application in a prolonged intermittent fashion (extended daily hemofiltration (EDHF)). After accounting for relevant confounding variables in multivariable analysis, initial treatment with CVVHDF was associated with significant 3.8-fold higher odds of recovery of kidney function when compared with initial therapy with EDHF. This difference was evident despite the fact that patients receiving CVVHDF had significantly lower initial mean arterial pressures, more oliguria and lower serum pH at the time of RRT initiation. There was no difference in adjusted mortality rates between the two modalities.

The findings of Sun and colleagues are consistent with those obtained in several large cohort studies [2-4] in which higher rates of recovery to dialysis independence were found in survivors of critical illness complicated by AKI initially treated with continuous renal replacement therapy (CRRT) compared with those treated with intermittent renal replacement therapy (IRRT). In a systematic review including 50 studies reporting dialysis dependence in AKI survivors [5], IRRT as an initial modality was associated with a 1.7 times greater risk for dialysis dependence when compared with CRRT (odds ratio, 1.73; 95% confidence interval, 1.35 to 1.68). However, these results are susceptible to treatment allocation bias, as the effect was largely driven by observational studies and was nonsignificant when the analysis was restricted to randomized controlled trials (odds ratio, 1.15; 95% confidence interval, 0.73 to 1.68; *n* = 7). Recently, a large, population-based Canadian study (not included in the meta-analysis) similarly compared renal recovery among survivors of severe AKI according to the initial RRT modality and included a propensity matched analysis to adjust for treatment allocation [6]. In this study, initial treatment with IRRT, when compared with CRRT, was also associated with a significantly higher likelihood of dialysis dependence at 90 days (21% vs 16%) and during long-term follow-up (27% vs 21%).

One shortcoming of these studies was the fact that data were sourced from administrative databases or population registries and that these could not provide patient-level data. The study from Sun and colleagues therefore provides further insights and granularity on patient characteristics at the time of RRT initiation and details on treatment provision [1]. Their data provide plausible explanations for the higher rate of renal recovery associated with initial treatment with CRRT.

First, use of CRRT was associated with a trend for fewer episodes of hypotension (15% vs 26%, *P* = 0.112) and higher average mean arterial pressure during the first 72 hours of therapy (89.7 mmHg vs 83.8 mmHg, *P* = 0.137) when compared with IRRT. Although these differences failed to reach statistical significance, they were clinically important. This nonsignificance is presumably due to the higher delivered hourly ultrafiltration rate necessary with IRRT to achieve fluid homeostasis targets compared with CRRT (241 ml/hour in the EDHF group vs 149 ml/hour in the CVVHF group, *P* <0.001). Indeed, Conger and colleagues have long established the loss of autoregulation of renal blood flow in AKI, and the impact of hypotension contributes to further histological damage [7,8]. The increased occurrence of iatrogenic hypotension induced by IRRT to achieve fluid removal targets can logically be expected to contribute to delayed or reduced likelihood of renal recovery.

Second, despite a higher hourly ultrafiltration rate, the use of EDHF was associated with a lower ability to remove fluids in the first 72 hours. This is demonstrated by the net negative fluid balance obtained in the CVVHDF group but not in the EDHF group (–0.46 l vs +0.15 l, *P* = 0.019). The capacity to safely remove fluid in critically ill oligoanuric patients is limited in IRRT compared with CRRT. Fluid accumulation and overload are now recognized as important complications of critical illness and are associated with adverse outcomes [9,10] and reduced renal recovery [10,11]. A direct causal relationship between positive fluid balance and worse renal outcome has yet to be determined; however, these factors may be related to increased pressure exerted by extravascular fluid within an encapsulated organ [12].

Finally, the continuous application of RRT mathematically translated into almost double the delivered RRT dose during the first 72 hours (replacement flow: CVVHDF group 4.64 l/day vs EDHF group 2.65 l/day, *P* <0.001). Whether this higher and more consistently delivered dose early in a patient’s course of critically illness translates into better metabolic homeostasis remains speculative and should be explored in randomized trials, despite prior trials showing no significant impact on survival or recovery by delivered dose [13-15]. The role of enhanced clearance of inflammatory molecules on renal recovery remains to be evaluated.

Overall, the study by Sun and colleagues further contributes to the growing body of evidence to suggest that initial treatment with CRRT compared with IRRT in critically ill patients with AKI may confer superiority for increased likelihood of renal recovery. The physiologic reasoning for this conclusion is biologically plausible: CRRT is associated with better hemodynamic stability through reduced episodes of hypotension and improved fluid homeostasis. In the absence of a suitable powered randomized trial with renal recovery as a primary endpoint, the evidence supporting the superiority of initial treatment with CRRT with renal recovery in mind will be derived from observational data such as these. However, given the current burden of evidence suggesting better recovery with CRRT as the initial therapy, physicians should seriously consider moving away from potentially deleterious therapies before proof of their non-inferiority is established. While lower healthcare costs associated with intermittent hemodialysis are often advocated for their primary use in critically ill patients with AKI, formal economic analyses taking long-term dialysis costs into account are necessary to establish the real cost of IRRT in the ICU.

## Abbreviations

AKI: Acute kidney injury; CRRT: Continuous renal replacement therapy; CVVHDF: Continuous venovenous hemodiafiltration; EDHF: Extended daily hemofiltration; IRRT: Intermittent renal replacement therapy; RRT: Renal replacement therapy.

## Competing interests

SMB and AGS have consulted and received speaker fees from Gambro Renal Inc. (Baxter).
